# Tumour budding and its clinical implications in gastrointestinal cancers

**DOI:** 10.1038/s41416-020-0954-z

**Published:** 2020-06-30

**Authors:** Inti Zlobec, Martin D. Berger, Alessandro Lugli

**Affiliations:** 1 Institute of Pathology, University of Bern, Bern, Switzerland; 2 Department of Medical Oncology, Inselspital, University Hospital Bern, University of Bern, Bern, Switzerland

**Keywords:** Biomarkers, Oncogenesis

## Abstract

Tumour budding in colorectal cancer has become an important prognostic factor. Represented by single cells or small tumour cell clusters at the invasion front of the tumour mass, these tumour buds seem to reflect cells in a ‘hybrid’ state of epithelial–mesenchymal transition, and evidence indicates that the presence of these entities is associated with lymph node metastasis, local recurrence and distant metastatic disease. The International Tumour Budding Consensus Conference (ITBCC) has highlighted a scoring system for the reporting of tumour budding in colorectal cancer, as well as different clinical scenarios that could affect patient management. Other organs are not spared: tumour budding has been described in numerous gastrointestinal and non-gastrointestinal cancers. Here, we give an update on ITBCC validation studies in the context of colorectal cancer and the clinical implications of tumour budding throughout the upper gastrointestinal and pancreatico-biliary tract.

## Background

**Fig. 1:**
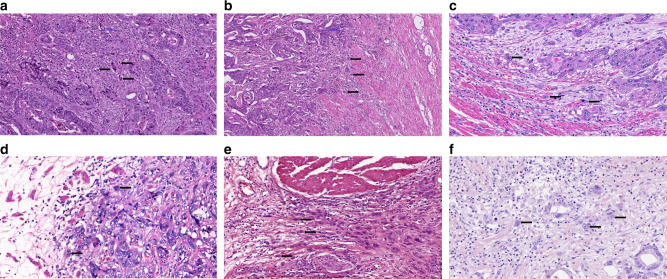
Tumour budding (visualised by arrows) in different gastrointestinal cancers. Intratumoural (ITB) (**a**) and peritumoral (PTB) (**b**) budding in colorectal cancer; tumour buds at the invasive front of oesophageal squamous cell cancer (**c**) and oesophageal adenocarcinoma (**d**), and tumour budding in gastric (**e**) and pancreatic ductal adenocarcinoma (**f**). Original images from cases at the Institute of Pathology, University of Bern, approved by the ethics committee of the canton of Bern (KEK Bern). Patients have signed written informed consent.

Interest in tumour budding and its clinical implications has surged over the past few years. Tumour budding is defined by the presence of single tumour cells or small clusters of cells within the tumour centre (‘intratumoural’ budding, ITB) (Fig. 1a) or at the tumour-invasion front (‘peritumoural’ budding, PTB) (Fig. 1b).^1^ As these entities can be distributed throughout the tumour mass, tumour budding is amenable to detection in surgical resections as well as by biopsy, which is potentially highly significant in the context of pre- and post-operative patient management for some tumour types. Tumour budding occurs in a large variety of cancers from different organs.^2^ The frequency of high-grade tumour budding is difficult to estimate, due to the use of various scoring systems, but it might be found in approximately 40% of colorectal cancers (CRC),^3^ oesophageal cancers and gastric cancers^4–8^ (Fig. 1c–e), as well as in more than 50% of pancreatic ductal adenocarcinomas (PDACs)^9^ (Fig. 1f) and cholangiocarcinomas.^10^ Tumour budding is also reported to occur in many other cancer types, such as head and neck cancers,^11^ lung adenocarcinomas and squamous cell carcinomas^12,13^ as well as breast^14^ and cervical^15^ cancers.

Evidence indicates that tumour buds might adopt the properties of cells undergoing epithelial–mesenchymal transition (EMT), suggesting that these cells have a more invasive and migratory potential.^16^ Double staining for the epithelial marker cytokeratin and the mesenchymal marker vimentin, highlights a small number of tumour buds that co-express both proteins, adding weight to the hypothesis that a ‘hybrid’ EMT phenotype exists in a subset of these cells.^17^ EMT has been linked to therapy resistance and cancer-cell stemness,^18,19^ so it follows that the detection of tumour budding in preoperative biopsy samples of patients with, for example, either rectal or oesophageal cancers, is associated with a poor response to neoadjuvant therapy and overall clinical outcome.^20,21^ Mounting data suggest that the presence of tumour budding is an unfavourable prognostic factor across all tumour types in which it is found and is tightly associated with lymph node metastasis, local recurrence and distant metastatic disease.

The reason for the occurrence of tumour budding is not known. DNA sequencing studies show no difference in the mutational profile of driver genes in tumour buds in comparison with the main tumour mass,^22^ although RNA sequencing studies clearly underline changes in mRNA and microRNAs involved in transforming growth factor-β (TGF-β) and WNT signalling pathways.^3,23^ Downstream of these pathways, repressors of the cell–cell adhesion molecule E-cadherin are overexpressed, as are markers of extracellular matrix degradation and migration. The presence of tumour buds in areas of desmoplastic stroma strongly suggests an interplay between tumour cells and cancer-associated fibroblasts, which presents an exciting area for future investigation.

Although first described in cancer of the lip,^24^ tumour budding has increased in popularity as an important prognostic factor in CRC. The first guideline for reporting tumour budding was published in 2017 for CRC following the 2016 International Tumour Budding Consensus Conference (ITBCC),^1^ and is now included in the College of American Pathologists (CAP) protocol. The ITBCC method was selected due to the large evidence base supporting its clinical utility, and is based on the Japanese Guidelines for Colorectal Cancer Reporting.^25^ The evidence-based guidelines describe clinical scenarios in which tumour budding should be assessed using a three-tier scoring system. In detail, the haematoxylin and eosin (H&E) slide with the greatest degree of budding at the invasion front (PTB) is selected, then ten individual fields at medium power (10× objective) are used to identify the ‘hotspot’ and tumour buds are counted using a ×20 objective. Normalisation of the count to an area of 0.785 mm^2^ (depending on the eyepiece field number) is performed, and the budding categories are defined: BD1 (1–4 buds), BD2 (5–9 buds, BD2) and BD3 (≥10 buds). Subsequent validation of these guidelines not only in CRC, but also in lung, gastric and PDAC, has underlined the usefulness of this standardised scoring approach.^12,26,27^ H&E is the standard stain, but pan-cytokeratin staining can also be used in conjunction with a number of approaches to assess tumour budding (Box 1).

In this review, we focus on presenting the latest data on budding in tumours of the gastrointestinal tract, giving an update on the clinical application of tumour budding in CRC and highlighting the latest data in the fields of oesophageal and gastric cancers, as well as PDAC and cholangiocarcinomas.

Box 1 Methods to assess tumour budding A variety of different methods have previously been used to assess tumour budding, both intra- and peri-tumourally using either pan-cytokeratin or H&E-stained slides.^97,98^ These range from focusing on a specific area or objective lens (×20 or ×40) followed by evaluation of buds in one or ten hotspots, to more subjective scoring methods based on the observed severity of the budding throughout the slide (e.g. low/moderate/severe). The actual tumour budding counts have been used in both pan-cytokeratin and H&E for analysis with clinical endpoints, and cut-offs for classification into ‘low’ or ‘high’ have been suggested at four, five or ten buds, depending on the clinical scenario and the type of material (biopsy sample or resection). Both have advantages and disadvantages. On the one hand, pan-cytokeratin helps to facilitate the visual count and results in a higher number of true tumour buds being detected, but also might lead to overestimation of non-bud-type objects, such as cell fragments. The nucleus, which needs to be observed to classify a cell as a bud, can be masked by strong cytokeratin staining. These issues contribute to interobserver variability, especially on a single-object level.^95^ H&E is a standard staining method and can be implemented in all pathology laboratories, but leads to difficulties in areas of peritumoural inflammation, and is challenging for the discrimination of tumour budding versus activated fibroblasts—again, a source of interobserver variation.

## Clinical implications of tumour budding in CRC

In the era of personalised healthcare, the role of biomarkers is of immense importance. The ideal biomarker is prognostic and/or predictive, simple, reproducible and cost-effective and, therefore, only rarely are all these requirements fulfilled. Molecular biomarkers of the Ras signalling pathway, such as KRAS, HRAS, NRAS and BRAF, have been shown to play an important role in the pathogenesis of CRC;^28^ however, it should be kept in mind that the sum of all the molecular features probably leads to a particular morphological picture that has defined histopathological characteristics. One of those histological characteristics in CRC is tumour budding, a phenomenon that is indicative of tumour progression and adverse prognosis.^29^ There are now enough data in the literature, as well as the ITBCC guidelines, to justify implementing the assessment of tumour budding into routine clinical practice for CRC.^30^ In CRC, the presence of tumour budding along with other established biomarkers might support clinicians in four potential clinical scenarios (summarised in Table 1). First, PTB might indicate which patients benefit from oncological resection after the diagnosis of a primary tumour that has grown into the submucosa (pT1 CRC). Second, PTB in stage II CRC might indicate patients who should be considered for adjuvant therapy. Third, ITB can also be assessed in biopsy samples and therefore be included in the preoperative management, especially of rectal cancer patients who might undergo a neoadjuvant therapy. Fourth, in stage IV CRC patients, the presence of intrametastatic or perimetastatic tumour budding (IMB and PMB, respectively) in colorectal cancer liver metastases (CRLM) might be a supportive marker to stratify patients for different therapeutic options. The utility of tumour budding in patients with stage III cancers has not been evaluated in depth. However, as adjuvant therapy is the standard-of-care, the question arises as to whether tumour budding might be predictive of the response to chemotherapy in this subgroup of patients.

**Table 1:** Clinical scenarios of tumour budding in colorectal cancer.

Clinical scenario	ITBCC recommendations	Clinical implication	Prognostic value	Predictive value
pT1 CRC	Tumour budding is an independent predictor of lymph node metastasis in pT1 CRC	Oncologic resection	Yes	Unclear, validation needed
Stage II CRC	Tumour budding is an independent predictor of survival in stage II CRC	Adjuvant therapy	Yes	Unclear, validation needed
ITB in preoperative biopsies	ITB in CRC has been shown to be related to lymph node metastasis	Neoadjuvant therapy in rectal cancer Preoperative surgical management in colon cancer	Yes	Possible, validation needed
Tumour budding CRLM	No recommendation	Additional factor for management of stage IV CRC patients	Possible, validation needed	Unclear, validation needed

*ITB* ‘intratumoural’ budding, *CRC* colorectal cancer, *CRLM* colorectal cancer liver metastases.

### Clinical scenario 1: tumour budding in pT1 CRC

The clinical management of pT1 CRC includes the decision as to whether patients with early invasive cancer should undergo a wait-and-see approach or if they should be considered for oncological resection. There is therefore a major need for robust and reproducible biomarkers that correlate with the presence or absence of lymph node metastases. In 2004, Ueno et al. investigated a panel of clinicopathological parameters, including tumour location, tumour diameter, macroscopic tumour configuration (sessile vs. pedunculated), tumour grade, vascular invasion, tumour budding and width and depth of submucosal invasion.^31^ The study concluded that the absence of a number of features—including high tumour grade, vascular invasion, budding and extensive submucosal invasion—might potentially favour a watch-and-see policy.^31^ In 2013, Bosch et al. obtained similar results from a meta-analysis of 17 studies and 3782 patients with pT1 CRC.^32^ The results showed strong predictors for lymph node positivity to be submucosal invasion ≥1 mm (relative risk [RR] 5.2, 95% confidence interval [CI] 1.8–15.4), lymphatic invasion (RR 5.2, 95% CI 4.0–6.8), poor histological differentiation (RR 4.8, 95% CI 3.3–6.9) and tumour budding (RR 5.1, 95% CI 3.6–7.3). The conclusion was therefore quite similar to that proposed by Ueno et al.^31^—namely, that the absence of lymphatic invasion and budding, submucosal invasion ≤1 mm and poor histological differentiation was each associated with a low risk of lymph node metastases.^32^ In 2017, Cappellesso et al. focused specifically on the role of tumour budding in a meta-analysis of 41 studies and 10137 patients with pT1 CRC.^33^ They found tumour budding to be strongly associated with the risk of nodal metastases and, when comparing a positive tumour-budding status (684/2401, 28.5%) with a negative tumour-budding status (557/7736, 7.2%), the prevalence of lymph node positivity resulted in an odds ratio (OR) value of 6.44 (95% CI, 5.26–7.87, *P* = 0.0001; I2 = 30%, 41 studies).^33^ The ITBCC states that tumour budding is an independent predictor of lymph node metastases in pT1 CRC patients, and therefore strongly recommends that tumour budding is reported, along with other histopathological predictors of lymph node metastasis, such as poor differentiation, lymphovascular invasion and the depth/level of submucosal invasion,^1^ in patients with pT1 CRC.

### Clinical scenario 2: tumour budding in stage II CRC

The updated European Society for Medical Oncology (ESMO) guidelines for the management and treatment recommends a follow-up for patients with low-risk stage II colon cancer, while adjuvant therapy with fluoropyrimidine should be considered for patients who have high-risk factors, such as T4 (the tumour has grown through all layers of the colon and attached to or invaded other structures and organs), number of examined lymph nodes <12, primary tumour perforation or occlusion, tumour grade 3 or absence of microsatellite instability (MSI).^34,35^ In the past 10 years, numerous studies and meta-analyses have reported tumour budding to be an independent factor of poor survival and recurrence in patients with stage II CRC, with outcomes similar to those of patients with stage III CRC.^36–44^ The ITBCC therefore recommended in 2016 that tumour budding be included among the high-risk factors in stage II CRC.^1^ This recommendation was supported by the 2019 World Health Organisation (WHO) classification of tumours of the digestive system, which reports tumour budding—along with perineural invasion, intramural and extramural vascular invasion, lymphatic invasion and tumour deposits—as a high-risk factor with an OR of 4.51 (95% CI, 2.55–7.99).^45^ In 2019, Ueno et al. validated the ITBCC scoring system in a multicentre stage II colon cancer cohort from 123 institutions (*n* = 991).^46^ The 5-year relapse-free survival (RFS) rate was 90.9% in patients with tumours classified as BD1, 85.1% in those with BD2 and 74.4% in those with BD3 (*P* < 0.001). There also was a significant correlation between the budding grade and recurrence in the liver, lungs, lymph nodes and peritoneum (*P* < 0.001–0.01). Multivariable analysis revealed that budding had an independent impact on RFS. The study concluded that tumour budding should be routinely reported in stage II colon cancer.^46^

### Clinical scenario 3: tumour budding in CRC preoperative biopsy samples

In 1989, Morodomi et al. described the presence of tumour buds in biopsy samples from patients with rectal cancer and the association of this phenomenon with lymph node metastases.^47^ This observation led to a systematic assessment of PTB and ITB,^48^ and to the finding of a potential prognostic and predictive role for ITB. ITB is highly associated with PTB, and therefore a surrogate for the tumour-budding status of the whole tumour, as well as being associated with lymph node and distant metastases, local recurrence, poor survival and tumour regression grade.^49–53^ Therefore, the assessment of ITB in biopsy samples might have important clinical implications, especially in the preoperative management of rectal cancer patients. Patients who present with high-grade ITB along with the already-implemented clinical factors in preoperative biopsy samples might be considered for neoadjuvant therapy. Although the ITBCC recognises the ITB approach, more data are definitely necessary prior to its implementation in daily practice.^1^

### Clinical scenario 4: tumour budding in CRLM

The ESMO consensus guidelines for the management of patients with metastatic CRC highlight the importance of a multidisciplinary management, including oncology, surgery, radiology and pathology.^54^ The most frequently used biomarkers in clinical practice are molecular, and report on the RAS, BRAF and MSI status,^54^ whereas current histopathological features reported for clinical management are metastatic size, percentage of fibrosis and necrosis, resection status and tumour regression grade.^55^ Several studies have shown the prognostic potential of the histological growth pattern—desmoplastic, pushing and replacement—of the tumour–liver interface of CRLM.^56–62^ Tumour budding, a morphological feature of the tumour microenvironment at the invasive front, might therefore also be an important factor in disease progression in stage IV CRC patients. Similar to the primary tumour, tumour buds can be detected at the invasive front (PMB) or within the main metastasis body (IMB)^63^ but, in contrast to the primary tumour, there is still a major challenge for scoring tumour budding in CRLM. Indeed, the detection of tumour buds can be difficult in cases without desmoplastic stromal reaction or a strong reactive perimetastatic ductular proliferation.^63^ In addition, pan-cytokeratin immunohistochemical staining can sometimes obscure important morphological features, and therefore a budding score based on H&E staining is recommended.^63^ In a 2018 study, tumour budding assessed in CRLM from 229 patients with stage IV CRC was a prognostic factor, but not an independent predictor of survival.^64^

In summary, there are currently not enough data to make any firm conclusions on the prognostic or predictive role of tumour budding in CRLM, and further retrospective and prospective studies on large multicentric cohorts are needed.

## Clinical implications of tumour budding in oesophageal and gastric cancer

The first study on tumour budding in oesophageal squamous cell carcinoma (SCC) dates from the early 2000s. Investigating tumour budding by H&E in a small cohort of 56 patients, which included surgically treated individuals with stage I–III oesophageal SCC, Roh et al. found a marked reduction in the 3-year survival rate in patients with high-grade versus low-grade budding (30.7% vs. 72.3%, respectively).^65^ Similar results have been observed in numerous studies (Koike et al.,^66^ Miyata et al.^21^ and Teramoto et al.,^67^ Jesinghaus et al.,^68,69^ Niwa et al.^70^ and Ito et al.^71^) underlining significantly poorer 3-year or 5-year survival rates after oesophagectomy in SCC patients with high-grade budding. Nakanishi and colleagues published comparable results, with 5-year survival rates of 49% versus 15% in 74 patients with low-grade versus high-grade budding, respectively, receiving preoperative chemotherapy.^72^ The results of these studies have been reviewed by Koelzer et al.^73^ High-grade tumour budding has also been found to be associated with lymph node metastasis in oesophageal lesions involving the muscularis mucosae (T1a-MM) to those of the upper third of the submucosa (T1b-SM1) using both H&E and cytokeratin staining.^74^ These results suggest that budding could be a useful histomorphological feature in patients with primary resected oesophageal SCC in the neoadjuvant setting and in early-stage cancers.

Although only a handful of studies have evaluated tumour budding in oesophageal adenocarcinoma, similar results have been reported.^73^ High-grade tumour budding is described in 28–51.7% of cases, albeit using different scoring systems and based on either H&E or pan-cytokeratin staining.^75^ Its presence is associated with higher TNM stage, lymph node metastases, poor disease-free survival and poor overall survival. These results again highlight the correlation between tumour budding and disease course, and might be useful to guide follow-up.

Guo and colleagues published a review summarising the evidence of tumour budding in gastric cancer in 2019.^7^ Seven cohorts encompassing data from 2178 patients were analysed; the method used to analyse budding was based on H&E staining, and the cut-off values for ‘high-grade’ budding varied across studies, from five or ten buds to the median number of buds in the particular cohort. As a first step, the presence of high-grade tumour budding showed a positive correlation with tumour stage (OR 6.63, 95% CI 4.01–10.98, *P* < 0.0001) as well as with undifferentiated tumour status (OR 3.74, 95% CI 2.68–5.22, *P* < 0.01). High-grade tumour budding was significantly associated with lymphatic vessel invasion and lymph node metastasis (OR 7.85, 95% CI 5.04–12.21, *P* < 0.01, and OR 5.75, 95% CI 3.20–10.32, *P* < 0.01, respectively), as well as with poor 5-year overall survival in a pooled analysis of 1833 patients (HR 1.79, 95% CI 1.53–2.05, *P* < 0.01). These results were confirmed in a subgroup analysis of intestinal-type cancers but not in diffuse-type adenocarcinoma.^76^ In their evaluation of tumour budding in 621 radical gastrectomies for submucosal early gastric carcinoma,^77^ Du et al. identified high-grade tumour budding as a predictor of lymph node metastases (OR 3.3, 95% CI 1.9–5.9). Moreover, when Ulase et al. applied the ITBCC tumour-budding score to 456 surgically resected gastric cancers, they found that the BD score was significantly associated with sex, Laurén phenotype, pT-, pN- and pM classification, as well as perineural invasion and survival times.^26^

In summary, tumour budding shows prognostic potential in oesophageal adenocarcinomas, SCC and gastric cancers, and might be predictive in the neoadjuvant setting. Although a standardised scoring system is still missing, the ITBCC approach for CRC might also be applicable in the context of upper gastrointestinal cancers.

## Clinical implications of tumour budding in cholangiocarcinoma and pancreatic ductal adenocarcinoma

Cholangiocarcinoma is a rare type of tumour, comprising <1% of all cancers;^78^ it is subdivided into intrahepatic and extrahepatic cholangiocarcinoma, and the latter can be further subclassified as perihilar or distal cholangiocarcinoma.^79^ The vast majority of patients with cholangiocarcinoma present with unresectable disease at the time of diagnosis. Consequently, the prognosis for cholangiocarcinoma is poor, with a 5-year OS of 30–40% for localised tumours and a median OS of nearly 12 months for unresectable or metastatic disease.^80,81^

Although tumour budding is a well-established prognostic factor in CRC,^82^ its significance in cholangiocarcinoma is far less clear. However, Ogino et al. demonstrated in 2019 that peritumoral budding in both perihilar and extrahepatic cholangiocarcinoma is associated with adverse clinicopathological features, such as higher T stage, lymphovascular and perineural invasion, lymph node metastases and higher histological grade, which translate into a worse clinical outcome.^83^ Cholangiocarcinoma patients with high-grade tumour budding had a significantly shorter OS compared with those with low-grade tumour budding. In another cholangiocarcinoma cohort comprising 299 Asian patients, the presence of peritumoral budding was associated with worse OS.^84^ According to the results of these two retrospective studies, tumour budding might be a potential prognostic factor.

Based on the results of the adjusted intention-to-treat and per-protocol analysis of the previously published BILCAP trial, a randomised clinical trial comparing adjuvant chemotherapy with capecitabine with expectant treatment following resection, the American Society of Clinical Oncology (ASCO) guidelines recommended adjuvant treatment with capecitabine in resected cholangiocarcinoma patients.^85,86^ However, the study population was heterogeneous, comprising all T and N stages with or without R0 resection. A preplanned subanalysis indicated that male patients and those with poorly differentiated tumours derived the most benefit from adjuvant capecitabine treatment,^83,85^ highlighting the importance of identifying predictive biomarkers to predict which subset of patients with resected cholangiocarcinoma should respond to chemotherapy. Further validation in independent datasets—preferably from Phase 3 studies—is needed to finally confirm both the prognostic and predictive values of tumour budding in cholangiocarcinoma. In addition, no data demonstrating whether patients with high-grade, tumour-budding cholangiocarcinoma might benefit from adjuvant chemotherapy exist yet. Therefore, the predictive impact of tumour budding in cholangiocarcinonoma is still unclear.

PDAC also has a poor prognosis, with a 5-year OS of ~5%.^87^ In 2019, a meta-analysis demonstrated that patients with PDAC exhibiting high-grade tumour budding had a higher all-cause mortality rate compared with those who showed low-grade tumour budding (HR 2.65, 95% CI 1.79–3.91, *P* < 0.0001).^88^ Due to the aggressive tumour biology and the inherent poor prognosis of PDAC, adjuvant chemotherapy with either FOLFIRINOX (folinic acid, fluorouracil, irinotecan and oxaliplatin) or gemcitabine combined with capecitabine is recommended in all patients after curative resected PDAC, regardless of the pathological stage.^89,90^ However, patients who may not qualify for a doublet or triplet therapy, treatment with gemcitabine alone is a reasonable option. In a retrospective analysis of the CONKO-001 trial, designed to compare adjuvant gemcitabine with observation in patients with PDAC undergoing complete, curative-intent tumour resection, the presence of tumour budding was associated with decreased OS, irrespective of whether or not the patients were treated with adjuvant gemcitabine.^91^ In this study, no further subclassification into PTB or ITB has been carried out. In contrast to the situation for stage II colon cancer, in which, although still unproven, the presence of high-grade tumour budding might contribute to the treatment strategy, tumour budding in PDAC has not yet had an impact on adjuvant treatment decisions. However, just as new treatment strategies targeting PDAC might continue to evolve in the near future, the role of tumour budding on clinical decision-making might be redefined.

## Perspectives for tumour budding in gastrointestinal cancers

Tumour budding is emerging as a promising morphological biomarker not only in CRC, but also in other gastrointestinal cancers, such as oesophageal, gastric, PDAC and cholangiocarcinomas^2,73,83^ (the prognostic and/or predictive value of tumour budding in non-CRC gastrointestinal cancers is summarised in Table 2). An interesting observation is the fact that tumour budding can be detected in adenocarcinomas and in SCCs, as these morphological tumour subtypes have different criteria for dedifferentiation (solid areas vs. keratinisation), which is highlighted by many studies investigating the clinical implication of tumour budding in oral cavity cancers.^92^ Nevertheless, one should keep in mind that, depending on the tumour type, the definition and scoring system for tumour budding might differ, similar to the tumour gradings published by the Union for International Cancer Control (UICC), American Joint Committee on Cancer (AJCC) and the WHO. Grading in SCC of the oesophagus is based on the degree of cytological atypia, mitotic activity and the presence of keratinisation, whereas in colorectal adenocarcinoma, grading is determined according to solid areas.

**Table 2:** Prognostic and predictive value of tumour budding in non-CRC gastrointestinal cancers.

	Reference	Year	Ethnicity	Design	Stage	Size	Cut-off*	Systemic therapy^♯^	Outcome	Prognostic value	Predictive value
*Oesophageal cancer*											
AC	Thies et al.^6^	2016	Caucasian	Retrospective	I–IV	200	5	n.a.	High versus low ITB associated with shorter OS (*P* = 0.029)	Yes	No
	Landau et al.^75^	2014	n.a.	Retrospective	I	210	3	n.a.	High versus low TB 5-y OS: 37% versus 71% (*P* < 0.0001)	Yes	No
SCC	Jesinghaus et al.^68^	2017	Caucasian	Retrospective	I–IV	135	15	n.a.	High versus low versus no TB mean OS: 39.1 versus 64.7 versus 140.8 months (*P* < 0.001)	Yes	No
	Niwa et al.^70^	2014	Asian	Retrospective	I–IV	78	3	n.a.	High versus low TB 5-y OS: 25.9% versus 75.1%, HR 5.33, 95% CI, 2.55–12.5, (*P* < 0.0001)	Yes	No
	Teramoto et al.^67^	2013	Asian	Retrospective	I, II, IV	79	3	n.a.	High versus low TB 3-y OS: 48.8% versus 94.5% (*P* < 0.001)	Yes	No
	Ito et al.^71^	2012	Asian	Retrospective	I–III	87	5	n.a.	Pos. versus neg. TB associated with shorter OS (*P* = 0.006)	Yes	No
	Nakanishi et al.^72^	2011	Asian	Retrospective	I–IV	82	5	n.a.	High versus low TB mOS: 31 versus 113 months (*P* = 0.0002)	Yes	No
	Miyata et al.^21^	2009	Asian	Retrospective	I–IV	74	5	Neoadjuvant chemotherapy with cisplatin, doxorubicin and 5-Fu	High versus low TB 5-y OS: 17% versus 49% (*P* < 0.001)	Yes	Unclear, validation needed
	Koike et al.^66^	2008	Asian	Retrospective	I–IV	136	5	n.a.	High versus low TB 5-y OS: 35.4% versus 81.3% (*P* < 0.001)	Yes	No
	Roh et al.^65^	2004	Asian	Retrospective	I–III	56	5	n.a.	High versus low TB 3-y OS: 30.7% versus 72.3% (*P* = 0.04)	Yes	No
*Gastric cancer*											
AC	Kemi et al.^76^	2019	Caucasian	Retrospective	I–IV	583	10	n.a.	High versus low TB 5-y OS: 23% versus 41%, HR 1.46, 95% CI 1.22–1.75 (*P* < 0.001)	Yes	No
	Che et al.^99^	2017	Asian	Retrospective	I–IV	296	5	n.a.	High versus low TB associated with shorter OS HR 2.260, 95% CI 1.617–3.159, (*P* < 0.001)	Yes	No
	Tanaka et al.^100^	2014	Asian	Retrospective	I–IV	153	10	n.a.	High versus low TB associated with shorter OS HR 1.61, 95% CI 1.12–2.41, (*P* = 0.0104)	Yes	No
	Gabbert et al.^101^	1992	Caucasian	Retrospective	I–IV	445	5	n.a.	High versus low TB mOS 15 versus 31 months (*P* < 0.0001)	Yes	No
*Cholangiocarcinoma*											
	Tanaka et al.^10^	2019	Asian	Retrospective	I–IV	201	5	n.a.	Pos. versus neg. TB ICC: mOS 18.9 versus 106 months, HR 4.21, 95% CI 2.45–7.23 (*P* < 0.001) PHCC: HR 3.46, 95% CI 1.57–7.62 (*P* = 0.002) ECC: HR 3.07, 95% CI 1.0–9.45 (*P* = 0.050)	Yes	No
	Ogino et al.^83^	2019	Asian	Retrospective	I–IV	310	DCC: 5 PHCC: 0–4, 5–11, ≥12	No chemotherapy	DCC: high versus low TB mOS: 40 versus 169 months (*P* < 0.001) PHCC: high versus intermediate versus low mOS: 38 versus 58 versus 142 months (*P* < 0.001)	Yes	No
	Okubo et al.^84^	2018	Asian	Retrospective	I–IV	299	5	n.a.	Pos. versus neg. TB mOS: 18.5 versus 55.1 months HR 2.14, 95% CI 1.25–3.68 (*P* < 0.01)	Yes	No
*Pancreatic ductal adenocarcinomas*											
	Lohneis et al.^91^	2018	Caucasian	Retrospective	I–III	162	n.a.	Gemcitabine versus observation	High versus low TB OS: HR 1.040, 95% CI 1.019–1.061 (*P* < 0.001)	Yes	Unclear, validation needed
	Kohler et al.^102^	2015	Caucasian	Retrospective	I–IV	108	10	n.a.	High TB not associated with long-term survival (*P* = 0.073)	Yes	No
	O’Connor et al.^103^	2015	Caucasian	Retrospective	I–III	192	10	n.a.	High versus low TB mDSS 1.24 versus 1.42 years (*P* = 0.302) TB+ versus TB–associated with shorter mDSS (*P* = 0.0003)	Yes	No
	Karamitopoulou et al.^104^	2013	Caucasian	Retrospective	I–IV	117	10	n.a.	High versus low TB associated with decreased OS (*P* < 0.0001)	Yes	No

*OS* overall survival, *CI* confidence interval, *HR* hazard ratio, *PHCC* perihilar cholangiocarcinoma, *ECC* extrahepatic cholangiocarcinoma, *DCC* distal cholangiocarcinoma.

*Number of tumour buds; ^♯^information about chemotherapeutic drugs; TB, tumour budding.

For CRC, four aspects of tumour budding need to be addressed in order to optimise its clinical application. Interobserver variability in the assessment of budding is still suboptimal, especially among non-gastrointestinal pathologists, leading potentially to the up- or downgrading of budding. Accordingly, tumour-budding courses are needed to improve the reproducibility of tumour budding in CRC, similar to the assessment by immunohistochemistry of programmed death ligand 1 (PD-L1) in lung cancer.^30,93,94^ Alternatively, the development of a digitally supported scoring system for tumour budding could be time-saving as well as beneficial for increasing reproducibility.^95,96^ In addition, although enough data exist for tumour budding in pT1 CRC and stage II CRC patients for implementation into clinical practice, more studies are needed for the clinical scenarios that involve preoperative biopsies and the assessment of tumour budding in CRLM. Finally, although the predictive value of tumour budding is still not clear, the investigation of potential target molecules expressed by tumour buds might offer a promising approach for an anti-budding therapy to specifically target the tumour cells that seem to be responsible for local and distant metastases and consequently for tumour progression and decreased survival.

## Data Availability

All data are included in this published article.
